# Hospitals with and without neurosurgery: a comparative study evaluating the outcome of patients with traumatic brain injury

**DOI:** 10.1186/s13049-021-00959-2

**Published:** 2021-11-02

**Authors:** Aimone Giugni, Lorenzo Gamberini, Greta Carrara, Luca Antiga, Obou Brissy, Virginia Buldini, Italo Calamai, Akos Csomos, Alessandra De Luca, Enrico Ferri, Joanne M. Fleming, Primoz Gradisek, Rafael Kaps, Theodoros Kyprianou, Silvia Lagomarsino, Isaac Lazar, Costanza Martino, Malgorzata Mikaszewska-Sokolewicz, Andrea Montis, Gabor Nardai, Giovanni Nattino, Giuseppe Nattino, Giulia Paci, Laila Portolani, Nektaria Xirouchaki, Arturo Chieregato, Guido Bertolini, for The CREACTIVE consortium, Sárkány Ágnes, Fulvio Agostini, Claudio Ajmone-Cat, Giovanni Bassi, Vasileios Bekos, Marzia Bellin, Maria Grazia Bocci, Valeria Bonato, Alfeo Bonato, Manuela Bonizzoli, Paola Bonucci, Andrea Bottazzi, Giuseppe Calicchio, Fabrizia Carlin, Sergio Casagli, Carlo Alberto Castioni, Rita Ciceri, Francesco Cocciolo, Ezio Crestan, Peter Cseplo, Akos Csomos, Francesco Curto, Wojciech Dąbrowski, Anna De Cristofaro, Alessandra De Luca, Izabela Duda, Or Duek, Blanka Emoke Bakó, Nazzareno Fagoni, Paola Fassini, Enrico Ferri, Suada Filekovic, Gilberto Fiore, Emiliano Gamberini, Diego Gattari, Massimo Gianni, Maria Giovanna Dessena, Romano Giuntini, Stefania Guido, Rita Gyulai, Amir Hadash, Renata Hribar, Stavroula Ilia, Vesna Novak Jankovic, Vlado Jurekovic, Mateja Jereb, Maciej Kapias, Dragica Karadzic, Darja Kasnik, Anastasios Kioulpalis, Adrienn Kitti Szaszi, Janez Kompan, Eraclis Kyriakides, Sara Lamborghini, Sergio Livigni, Paolo Malacarne, Maria Martelli, Marina Alessandra Martin, Andrea Marudi, Martina Melis, Francesca Mengoli, Tomislav Mirkovic, Wiktoria Mizak, Marina Munari, Ennio Nascimben, Giuseppe Natalini, Giancarlo Negro, Csaba Nemes, Mara Olga Bernasconi, Michele Pagani, Vieri Parrini, Panagio Partala, Mauro Pastorelli, Isabella Pellicioli, Paolo Perino Bert, Nicola Petrucci, Simone Piva, Daniele Poole, Danilo Radrizzani, Anna Rekas, Paweł Robak, Antonio Rosano, Patrizia Ruggeri, Marco Sacchi, Mara Skoti, Alja Skrt, Ermanno Spagarino, Wiktor Sulkowski, Balázs Szedlák, Marina Terzitta, Rebecca Tinturini, Rossella Tofani, Paraskevi Tselioti, Ada Vecchiarelli, Elisabetta Venturini, Salvatore Visconti, Nektaria Xirouchaki, Valeria Zompanti, Roberto Zoppellari

**Affiliations:** 1 Anesthesia, Intensive Care and Prehospital Emergency, Maggiore Hospital, Bologna, Italy; 2 Laboratory of Clinical Epidemiology, Department of Public Health, Istituto Di Ricerche Farmacologiche Mario Negri IRCCS, Villa Camozzi, Via G.B. Camozzi 3, 24020 Ranica, Bergamo Italy; 3 Orobix, Bergamo, Italy; 4 Anesthesia and Intensive Care Unit, AUSL Toscana Centro, San Giuseppe Hospital, Empoli, Florence, Italy; 5 Hungarian Army Medical Center, Budapest, Hungary; 6 Neurointensive Care Unit, Department of Anesthesia and Intensive Care Unit, AOU Careggi, Florence, Italy; 7 Clinical Department of Anaesthesiology and Intensive Therapy, University Medical Centre Ljubljana, Ljubljana, Slovenia; 8 Faculty of Medicine, University of Ljubljana, Ljubljana, Slovenia; 9 General Hospital Novo Mesto, Novo Mesto, Slovenia; 10 University of Nicosia Medical School, Nicosia, Cyprus; 11 University Hospitals Bristol and Weston NHS Foundation Trust, Bristol, UK; 12 Pediatric Intensive Care Unit, Soroka Medical Center and The Faculty of Health Sciences, Ben-Gurion University of the Negev, Beer Sheva, Israel; 13 Anesthesia and Intensive Care Unit, AUSL Romagna, Maurizio Bufalini Hospital, Cesena, Italy; 14 Clinic of Anaesthesia and Intensive Care, Medical University of Warsaw, Warsaw, Poland; 15 Department of Neurorehabilitation, ASSL Oristano, ATS Sardegna, Oristano, Italy; 16 Department of Anaesthesiology and Intensive Care, Péterfy Hospital and Trauma Centre, Budapest, Hungary; 17 Intensive Care Unit, Azienda Socio Sanitaria Territoriale di Lecco, Lecco, Italy; 18 University Hospital of Heraklion, Crete, Greece; 19 Neurointensive Care Unit, Grande Ospedale Metropolitano Niguarda, Milan, Italy

**Keywords:** Brain injuries, Traumatic, Outcome assessment, GOS-E, Prospective studies, Rehabilitation

## Abstract

**Background:**

We leveraged the data of the international CREACTIVE consortium to investigate whether the outcome of traumatic brain injury (TBI) patients admitted to intensive care units (ICU) in hospitals without on-site neurosurgical capabilities (no-NSH) would differ had the same patients been admitted to ICUs in hospitals with neurosurgical capabilities (NSH).

**Methods:**

The CREACTIVE observational study enrolled more than 8000 patients from 83 ICUs. Adult TBI patients admitted to no-NSH ICUs within 48 h of trauma were propensity-score matched 1:3 with patients admitted to NSH ICUs. The primary outcome was the 6-month extended Glasgow Outcome Scale (GOS-E), while secondary outcomes were ICU and hospital mortality.

**Results:**

A total of 232 patients, less than 5% of the eligible cohort, were admitted to no-NSH ICUs. Each of them was matched to 3 NSH patients, leading to a study sample of 928 TBI patients where the no-NSH and NSH groups were well-balanced with respect to all of the variables included into the propensity score. Patients admitted to no-NSH ICUs experienced significantly higher ICU and in-hospital mortality. Compared to the matched NSH ICU admissions, their 6-month GOS-E scores showed a significantly higher prevalence of upper good recovery for cases with mild TBI and low expected mortality risk at admission, along with a progressively higher incidence of poor outcomes with increased TBI severity and mortality risk.

**Conclusions:**

In our study, centralization of TBI patients significantly impacted short- and long-term outcomes. For TBI patients admitted to no-NSH centers, our results suggest that the least critically ill can effectively be managed in centers without neurosurgical capabilities. Conversely, the most complex patients would benefit from being treated in high-volume, neuro-oriented ICUs.

**Supplementary Information:**

The online version contains supplementary material available at 10.1186/s13049-021-00959-2.

## Background

Traumatic brain injury (TBI) is an important medical, public health, and social problem worldwide [1–4] and is increasingly recognized as a global health priority. Globally, in 2016, TBI caused 8.1 million years of life lived with disabilities [5].

A considerable subset of TBI patients fails to return to baseline functional status at or beyond 3 months post-injury [6]. In some patients, further improvement is seen even as late as 5 years after injury. Evaluation of short-term outcomes, as hospital mortality, runs the risk of only partially revealing the problems related to the sequelae of TBI. For this reason, it is essential to explore the disability phenomenon at least 6 months after injury [7].

Effective public health response to TBI requires programs devised to minimize adverse outcomes among the injured, including efforts to improve acute care and early management, and strategies to ensure patient access to appropriate care and services [8].

European networks for severe trauma patient care are mainly designed according to the hub-and-spoke model. Although a uniform standard of care for trauma center (TC) levels have not yet been defined across Europe, in general, level I TCs are highly specialized hospitals, level II TCs are regional hospitals with intermediate facilities, and level III TCs are local hospitals with low expertise in trauma care. The hub-and-spoke model involves all healthcare facilities operating in a region according to their expertise and resources, establishing the criteria for centralizing patients. A strict balance between the patient’s needs and the level of care available in the receiving facility is fundamental to ensure the optimal functioning of such inclusive systems. Previous studies have found improved prognosis of the patients in mature, inclusive trauma systems [9, 10].

In addition to the general organ function support related to extra-cranial lesions, TBI patients admitted to ICUs could require neurosurgical interventions and specific expertise in neurocritical care. Therefore, these patients are usually directly admitted from the field or transferred early to hospitals with neurosurgical capabilities (NSH). On the other hand, since neurosurgical procedures, such as craniotomy or invasive intracranial pressure monitoring, are performed in less than 5% of TBI patients [11], other characteristics of NSH are expected to benefit TBI patients, as the greater intensity of neuro-oriented critical care [12], adherence to treatment guidelines [13], admission to high-volume centers [14] and an early, personalized, rehabilitation approach [15].

Nonetheless, a recent Italian study reported that about 12% of all severe TBI patients were admitted to the nearest hospital without neurosurgery capabilities (no-NSH) [16].

Various aspects could affect the decision to centralize TBI patients to NSH, as coma depth, pupil abnormalities, and severity of lesions in other districts. Moreover, it is not clear from the literature whether all strata of TBI severity requiring intensive care for either cranial or extra-cranial lesions effectively benefit from centralization to NSH.

This study aimed at evaluating whether centralization to NSH ICUs could have a role in the short- and long-term functional outcomes of these patients, as measured by the 6-month Glasgow Outcome Scale Extended (GOS-E) [17].

Data were extracted from the database of the CREACTIVE (Collaborative Research on Acute Traumatic Brain Injury in Intensive Care Medicine in Europe) consortium. CREACTIVE is an international prospective observational study aimed at describing the epidemiology of TBI in Europe and improving the quality of care in the field (ClinicalTrials.gov identifier: NCT02004080).

## Methods

### Study cohort

The CREACTIVE consortium was funded by the European Seventh Framework Programme for Research and Technological Development and involved 7 countries: Cyprus, Greece, Hungary, Israel, Italy, Poland, and Slovenia [18]. All TBI patients admitted to one of the participating ICUs were eligible for the study. More than 8000 patients from 83 ICUs were enrolled over 5 years, starting in March 2014.

To address the research question, we considered the adult patients of the CREACTIVE cohort and selected those admitted to the ICU no later than two days after the trauma. We did not exclude patients subject to secondary transfers.

To address the lack of a uniform classification of TCs across 7 different countries, with different trauma system programming rules [19], we categorized the participating ICUs as NSH and no-NSH centers, depending on the in-hospital availability of neurosurgery facilities.

Data on structural and organizational characteristics of the hospitals were collected through an ad-hoc web form. Patients’ characteristics were collected using an electronic case report form (eCRF), including demographics, trauma characteristics, clinical conditions at emergency rescue team arrival and at admission to the emergency department and ICU, comorbidities, details of computed tomography (CT) scans in the first hours after trauma, and outcomes. The eCRF was built upon the existing PROSAFE framework, implementing several completeness and validy checks to guarantee the uniformity of the collected data [20].

The full lists of comorbidities and injuries considered in the data collection are provided in Additional file 1: Tables S1 and S2. In particular, to minimize the efforts required by the data collection, the eCRF only included traumatic lesions characterized by an Abbreviated Injury Scale (AIS) severity greater than 2.

The Ethics Committee Lazio 1 of the Azienda Ospedaliera San Camillo Forlanini, Rome, Italy, and the local ethics committees at the participating centers approved the study protocol. Written informed consent was obtained from patients or their legal representatives. Where national legislation so permitted and considering the study’s observational nature, a waived or delayed consent process was implemented in patients in coma or experiencing high-stress levels.

### Outcome

The primary outcome of the study was the six-month GOS-E, the most widely used scale to evaluate disability in TBI patients. GOS-E is divided into 8 levels: death, vegetative state, severe disability (lower and upper), moderate disability (lower and upper), and good recovery (lower and upper), and was assessed by telephone interview. The ICU nurses made the calls; they were trained in a dedicated two-day course and were blinded to the aim of this study. The secondary outcomes of the study were ICU and hospital mortality.

### Statistical analysis

The results are presented following the research reporting guidelines of the Strengthening the Reporting of Observational Studies in Epidemiology (STROBE) statement [21]. Mean and standard deviation or median and interquartile range were used to describe quantitative variables. Binary and categorical variables were described by counts and proportions.

We used a propensity-score-matched design to address the research question. The propensity score is the probability of being exposed to a treatment of interest given pre-treatment variables [22]. Under a set of technical assumptions, this methodology can estimate causal effects in observational studies by attempting to create, a posteriori, the balanced design resulting from a randomized trial [23]. The propensity score was estimated by logistic regression, with the binary variable indicating whether the patient was admitted to a no-NSH center being the response. The variables included in the model were identified by a panel of clinicians involved in the study (ArC, AG, EF, LG, VB) as the set of variables related to both admission to an NSH center and patient outcome, as suggested by the literature [23]. No-NSH patients were 1:3 matched to NSH patients on the propensity score through the two-group optimal matching algorithm [24, 25]. The goal was to identify NSH patients similar to no-NSH ones with respect to all of the propensity-score variables, which included several demographics, characteristics of the trauma and clinical conditions (the list is provided in Additional file 1: Table S3). The panel of clinicians considered particularly important the balance of pupillary response at the emergency department (ED), pre-ICU hypotension, and age. Thus, we forced the algorithm to match exactly on the first two factors and to match no-NSH patients to NSH patients differing at most by 5 years of age.

The matching quality was evaluated in terms of propensity score balance, using standardized mean differences [23]. Differences smaller than 0.1 are commonly considered negligible. In well-balanced matched samples, outcome analyses may proceed with unadjusted comparisons. We compared the proportion of subjects in the outcome groups with risk differences (with 95% confidence intervals [CI]) and performed chi-squared tests. Statistical software R, version 3.6.1, was used for the analyses.

## Results

**Fig. 1:**
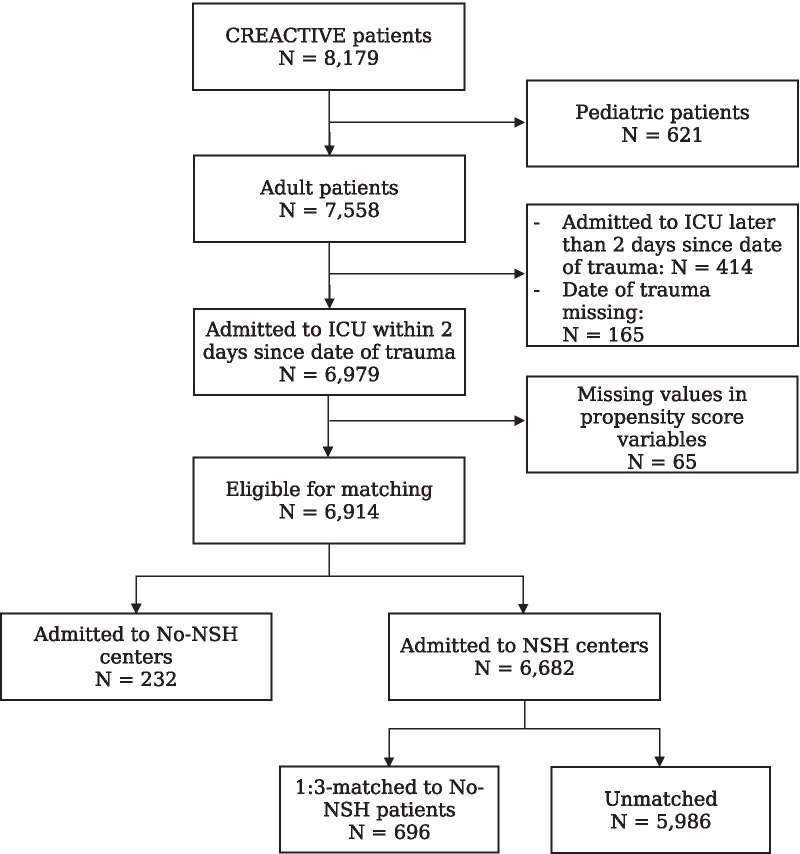
Flow-chart describing the patient selection

Figure 1 illustrates the flow of patients through the study. Of the 8179 CREACTIVE cohort patients, 7558 (92.2%) were adults. After excluding patients admitted to the ICU later than 2 days post-trauma and those with missing information, the cohort was formed by 6914 (91.4%) patients eligible for matching; of them, only 232 (3.4%) were admitted to ICUs in no-NSH hospitals. Additional file 1: Table S4 describes the countries where the participating ICUs were located and the structural/organizational characteristics of the ICUs belonging to the NSH, matched NSH, and no-NSH groups; Additional file 1: Figure S1 shows the number of subjects for each participating center.

The matched sample included a total of 67 ICUs: 14 no-NSH and 53 NSH. The no-NSH ICUs belonged to smaller hospitals (with a median of 333 vs. 664 beds), had a lower number of ICU beds (6 vs. 10), and managed a lower yearly number of both general trauma (28 vs. 77.9 patients/year) and TBI patients (9.8 vs. 47.6 patients/year).

A total of 24 variables were included in the propensity score model. The complete list of variables and the corresponding odds ratio estimates are provided in Additional file 1: Table S3. Following 1:3 propensity score-based matching, the final cohort included 928 patients: 232 admitted to the no-NSH ICUs and 696 matched from the NSH cohort.

Table 1 compares the demographic and clinical characteristics of the patients admitted to NSH and no-NSH ICUs before and after matching. Globally, compared to the cases admitted to no-NSH ICUs, the NSH cohort exhibited a lower prevalence of low-energy impacts and patients aged over 65, more severe derangements in terms of vital parameters both in the ED and at ICU admission, and worse Marshall grades at head CT scans.

**Table 1:** Demographic and clinical characteristics of the enrolled patients

Variables	NSH		No NSH	*P* value^a^
	All	Matched (1:3)		
N	6682	696	232	
Age				0.959
Mean (SD)	55.0 (21.0)	64.5 (20.1)	64.6 (20.3)	
Median (Q1–Q3)	57.0 (38.0–74.0)	71.0 (51.0–80.0)	73.0 (51.8–80.0)	
Sex (female)—N (%)	1775 (26.6)	213 (30.6)	71 (30.6)	1.000
Occupational status—N (%)				0.013
Worker	1633 (28.6)	120 (20.5)	38 (21.2)	
Retired	1594 (28.0)	265 (45.2)	102 (57.0)	
Unemployed	247 (4.3)	16 (2.7)	6 (3.4)	
Student	275 (4.8)	10 (1.7)	4 (2.2)	
Disabled/Sheltered employment	104 (1.8)	11 (1.9)	3 (1.7)	
Unknown	1849 (32.4)	164 (28.0)	26 (14.5)	
Missing^b^	980	110	53	
Marital status—N (%)				0.055
Unmarried/single	1204 (21.1)	79 (13.5)	21 (11.7)	
Married	2033 (35.6)	225 (38.4)	88 (49.2)	
Separated/divorced	172 (3.0)	16 (2.7)	8 (4.5)	
Widowed	399 (7.0)	69 (11.8)	17 (9.5)	
Unknown	1896 (33.2)	197 (33.6)	45 (25.1)	
Missing^b^	978	110	53	
Education level—N (%)				0.015
No schooling	117 (2.1)	13 (2.2)	4 (2.2)	
Primary school/elementary school	1230 (21.6)	133 (22.7)	63 (35.2)	
High school diploma	1043 (18.3)	81 (13.8)	19 (10.6)	
University degree or higher	243 (4.3)	16 (2.7)	2 (1.1)	
Unknown	3071 (53.8)	343 (58.5)	91 (50.8)	
Missing^b^	978	110	53	
Comorbidities—N (%)				
Any comorbidity^c^	3506 (52.5)	458 (65.8)	154 (66.4)	0.873
Antiplatelet therapy	517 (7.7)	63 (9.1)	20 (8.6)	0.842
Arrhythmia	608 (9.1)	108 (15.5)	36 (15.5)	1.000
Cerebrovascular disease	449 (6.7)	74 (10.6)	19 (8.2)	0.283
COPD	262 (3.9)	37 (5.3)	14 (6.0)	0.678
Dementia	180 (2.7)	14 (2.0)	7 (3.0)	0.372
Drug-induced coagulopathy	370 (5.5)	67 (9.6)	20 (8.6)	0.649
Diabetes Type II	706 (10.6)	81 (11.6)	26 (11.2)	0.859
Hypertension	2083 (31.2)	316 (45.4)	102 (44.0)	0.703
Myocardial infarction	358 (5.4)	49 (7.0)	25 (10.8)	0.069
Penetrating trauma—N (%)	210 (3.1)	16 (2.3)	4 (1.7)	0.602
Traffic accident—N (%)	3052 (45.7)	257 (36.9)	84 (36.2)	0.844
Trauma dynamic—N (%)				0.493
High-energy impact	2833 (42.4)	232 (33.3)	68 (29.3)	
Low-energy impact	2418 (36.2)	330 (47.4)	119 (51.3)	
Other	1430 (21.4)	134 (19.3)	45 (19.4)	
Missing	1	0	0	
Pre-treatment GCS—N (%)				0.382
Mean (SD)	8.9 (4.4)	11.0 (4.3)	11.2 (4.3)	
Median (Q1-Q3)	8 (5–13)	13 (7 -15)	13 (8–15)	
3–8	3354 (50.2)	195 (28.0)	62 (26.7)	
9–13	1735 (26.0)	211 (30.3)	60 (25.9)	
14–15	1593 (23.8)	290 (41.7)	110 (47.4)	
Main lesion—N (%)				
Cerebral contusion/laceration	1715 (25.7)	125 (18.0)	40 (17.2)	0.804
Extradural/epidural haematoma	581 (8.7)	42 (6.0)	10 (4.3)	0.323
Traumatic subdural haematoma	2216 (33.2)	161 (23.1)	59 (25.4)	0.476
Intraparenchymal bleeding	554 (8.3)	138 (19.8)	47 (20.3)	0.887
Diffuse injury without oedema	422 (6.3)	19 (2.7)	5 (2.2)	0.633
Diffuse injury with oedema	301 (4.5)	18 (2.6)	5 (2.2)	0.715
Subarachnoid haemorrhage	755 (11.3)	171 (24.6)	60 (25.9)	0.693
Skull fracture	138 (2.1)	22 (3.2)	6 (2.6)	0.658
Injuries other than TBId—N (%)				
Isolate TBI	3595 (53.8)	398 (57.2)	123 (53.0)	0.268
Abdomen	652 (9.8)	35 (5.0)	10 (4.3)	0.659
Chest	2089 (31.3)	193 (27.7)	73 (31.5)	0.276
Pelvis, bones, joints and muscles	1458 (21.8)	154 (22.1)	42 (18.1)	0.194
Major vessels	176 (2.6)	14 (2.0)	1 (0.4)	0.134
Spine	1363 (20.4)	135 (19.4)	46 (19.8)	0.886
Other	17 (0.3)	2 (0.3)	0 (0.0)	1.000
Time between injury and ICU adm. (hours)				0.085
Mean (SD)	8.3 (10.2)	9.1 (12.0)	7.6 (9.3)	
Median (Q1-Q3)	4.8 (2.9–8.5)	4.5 (2.7–9.0)	5.0 (2.8–7.4)	
Missing	1232	119	17	
Pupils at ED arrival—N (%)				0.985
Bilaterally reactive/miotic	4776 (71.5)	583 (83.8)	195 (84.1)	
Unilaterally dilated/non-reactive	1044 (15.6)	54 (7.8)	19 (8.2)	
Bilaterally dilated/non-reactive	637 (9.5)	36 (5.2)	11 (3.0)	
Not available	225 (3.4)	23 (3.3)	7 (3.0)	
Hypotension—N (%)	990 (14.8)	57 (8.2)	19 (8.2)	1.000
Hypoxia—N (%)	1587 (23.8)	74 (10.6)	24 (10.3)	0.902
Haemorrhagic-hypovolemic shock on ICU admission—N (%)	702 (10.5)	31 (4.5)	10 (4.3)	0.927
Neurogenic shock on ICU admission—N (%)	523 (7.8)	27 (3.9)	10 (4.3)	0.771
SAPS II (worst 24 h from ICU admission)				0.877
< 41 [probability: 0.00–0.25]	2085 (46.3)	330 (56.7)	120 (57.4)	
41–51 [probability: 0.26–0.50]	925 (20.6)	89 (15.3)	31 (14.8)	
52–63 [probability: 0.51–0.75]	808 (18.0)	97 (16.7)	38 (18.2)	
> 63 [probability: 0.76–1.00]	682 (15.2)	66 (11.3)	20 (9.6)	
SAPSII not assessable	2182	114	23	
Worst CT scan of first 48 h in ICU—N (%)				
Marshall scale				0.943
Diffuse Injury I	730 (10.9)	159 (22.8)	55 (23.7)	
Diffuse Injury II	2339 (35.0)	291 (41.8)	91 (39.2)	
Diffuse Injury III	631 (9.4)	42 (6.0)	13 (5.6)	
Diffuse Injury IV	229 (3.4)	34 (4.9)	11 (4.7)	
Mass lesion (V or VI)	2753 (41.2)	170 (24.4)	62 (26.7)	
Midline shift > 5 mm	2150 (32.2)	139 (20.0)	51 (22.0)	0.511
Lesion volume > 25 ml	2217 (33.2)	148 (21.3)	54 (23.3)	0.520
Petechiae	2419 (36.2)	180 (25.9)	59 (25.4)	0.897
Cistern condition				0.622
Normal	3046 (45.6)	339 (48.7)	105 (45.3)	
Compressed or distorted	2541 (38.0)	182 (26.1)	67 (28.9)	
Absent	1095 (16.4)	175 (25.1)	60 (25.9)	

^a^*P* value comparing no-NSH and matched NSH patients

^b^The information is not available for patients enrolled in 2014

^c^The complete list of comorbidities is reported in the Additional file 1: Table S1

^d^The complete list of lesions considered in each body region is reported in the Additional file 1: Table S2

The standardized means of the variables included in the propensity score before and after matching are provided in Additional file 1: Table S5, while Additional file 1: Figure S2 shows the relative distribution of the propensity score before and after matching. Since all standardized means were less or equal to 0.1 after matching, the no-NSH and NSH groups were considered well-balanced for all of the variables of interest.

**Fig. 2:**
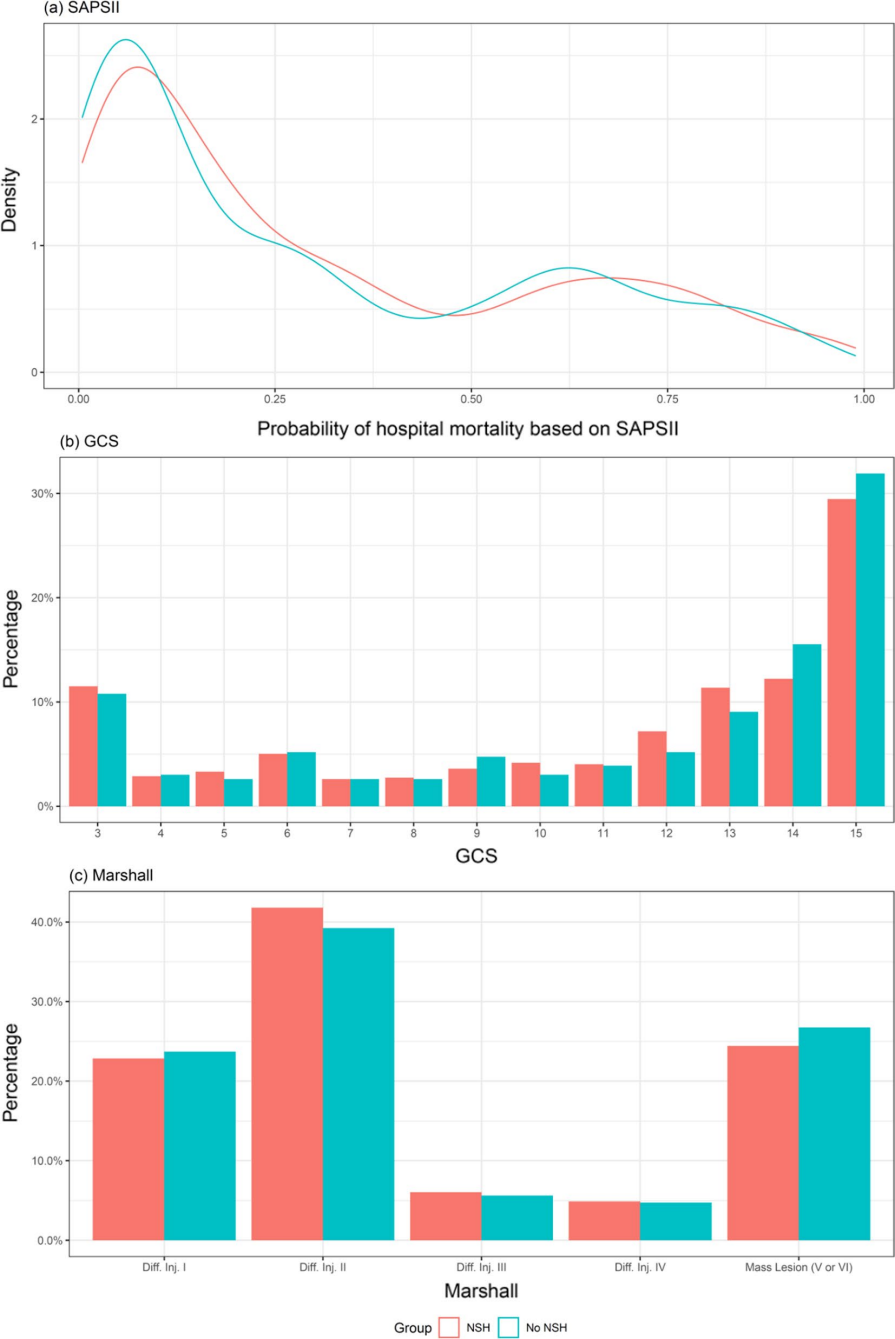
Distributions of the probability of hospital mortality as calculated by the SAPSII score (top panel), GCS before any treatment (central panel) and Marshall classification (bottom panel) in the NSH and no-NSH groups of the matched sample

Figure 2 depicts the distribution of matched patients’ cohorts among the predicted mortality strata, based on Simplified Acute Physiology score II (SAPS II) values at ICU admission,neurological status based on pre-treatment Glasgow Coma Scale (GCS) score and Marshall classification of the worst CT scan in the first 48 h in the ICU. Of note, pre-treatment GCS and Marshall classification were available for all patients while SAPS II score was unassessable at ICU admission for 137 out of 928 patients (14.8% of cases) due to deep sedation and failure to reliably estimate GCS within the first 24 h after ICU admission.

Clinical severity at ICU admission in the matched populations showed bimodal distribution. About half of the cases had SAPS II score below 40, corresponding to a predicted mortality lower than 25%, while a second peak was observed for SAPS II ranging from 52 to 63, with related expected mortality between 50 and 75%. As regards GCS evaluation pre-treatment, most patients fell in the range of mild TBI (GCS 13–15, 53.1% vs. 56.5%, respectively for NSH ICUs and no-NSH ICUs), while the second most represented group was classifiable as severe TBI (GCS 3–8, 28.0% vs. 26.8%). A similar bimodal pattern was observed for the Marshall CT classification. Further details can be found in Table 1.

Table 2 shows the outcome-related variables. Specifically, ICU and hospital mortality were significantly higher in the no-NSH group, with similar lengths of ICU and hospital stays. After ICU discharge, a slightly higher percentage of patients were directly transferred to rehabilitation wards in the NSH group.

**Table 2:** Interventions and outcomes of the study cohort

Variables	NSH		No NSH	*P* value^a^
	All	Matched (1:3)		
Interventions in ICUs—N (%)				
Hypothermia	93 (1.4)	5 (0.7)	1 (0.4)	0.636
External ventricular drainage without ICP monitoring	92 (1.4)	2 (0.3)	1 (0.4)	0.738
External ventricular drainage with ICP monitoring^b^	398 (6.0)	31 (4.5)	0 (0.0)	0.001
Barbiturate infusion for refractory ICP	393 (5.9)	18 (2.6)	1 (0.4)	0.045
Hyperventilation paCO2 < 25 mmHg	312 (4.7)	17 (2.4)	9 (3.9)	0.251
Indomethacin	23 (0.3)	0 (0.0)	0 (0.0)	1.000
Mannitol (multiple doses)	1613 (24.1)	119 (17.1)	43 (18.5)	0.618
Hypertonic saline	816 (12.2)	46 (6.6)	15 (6.5)	0.939
Invasive monitoring of intracranial pressure^c^	1930 (28.9)	105 (15.1)	6 (2.6)	< 0.001
Complications during ICU stay—N (%)				
Cardiovascular	693 (10.4)	59 (8.5)	14 (6.0)	0.231
Gastrointestinal	197 (2.9)	17 (2.4)	3 (1.3)	0.296
Neurological^d^	2438 (36.5)	179 (25.7)	74 (31.9)	0.067
Respiratory	954 (14.3)	90 (12.9)	12 (5.2)	0.001
Other	325 (4.9)	24 (3.4)	3 (1.3)	0.091
Infections	2423 (36.3)	193 (27.7)	44 (19.0)	0.008
Missing	2	0	0	
Three-day mortality—N deaths (%)	836 (12.5)	66 (9.5)	18 (7.8)	0.428
Missing	2	0	0	
*ICU outcome*				
Dead—N (%)	1569 (23.5)	143 (20.5)	67 (28.9)	0.009
Missing vital status	7	0	0	
If alive, conditions at discharge:				0.674
Follow simple commands—N (% on alive)	1443 (28.5)	116 (21.3)	32 (19.8)	
Cannot follow simple commands—N (% on alive)	3607 (71.5)	429 (78.7)	130 (80.2)	
Missing conditions at discharge	63	8	3	
If alive, discharged to:				< 0.001
Ward—N (% on alive)	2283 (44.7)	282 (51.0)	115 (69.7)	
Other ICU—N (% on alive)	1167 (22.9)	113 (20.4)	26 (15.8)	
High dependency unit—N (% on alive)	1276 (25.0)	137 (24.8)	16 (9.7)	
Rehabilitation—N (% on alive)	358 (7.0)	20 (3.6)	2 (1.2)	
Day hospital or long-term care—N (% on alive)	5 (0.1)	1 (0.2)	5 (3.0)	
Home—N (% on alive)	17 (0.3)	0 (0.0)	1 (0.6)	
Hospital mortality—N deaths (%)	2054 (30.9)	194 (28.0)	82 (35.5)	0.030
Missing	41	2	1	
ICU stay (days)—Median (Q1–Q3)				
Alive after ICU	9.0 (3.0–18.0)	5.0 (2.0–13.0)	4.0 (2.0–12.0)	0.872
Deaths in ICU	3.0 (1.0–7.0)	3.0 (1.0–7.0)	5.0 (2.0–10.0)	0.049
Missing	2	1	0	
Hospital stay (days)—Median (Q1–Q3)				
Alive after ICU	19.0 (10.0–34.0)	16.0 (9.0–.0)	14.0 (7.0–25.0)	0.130
Missing	17	0	0	
6-month GOS-E—N (%)				0.010
Severe disability pre-TBI	86 (1.6)	17 (3.2)	4 (2.1)	
Moderate disability pre-TBI	53 (1.0)	4 (0.7)	0 (0.0)	
Dead (1)	2308 (42.6)	228 (42.3)	87 (46.5)	
Vegetative State (2)	174 (3.2)	12 (2.2)	4 (2.1)	
Lower severe disability (3)	748 (13.8)	76 (14.1)	20 (10.7)	
Upper severe disability (4)	407 (7.5)	43 (8.0)	11 (5.9)	
Lower moderate disability (5)	267 (4.9)	21 (3.9)	2 (1.1)	
Upper moderate disability (6)	428 (7.9)	38 (7.1)	9 (4.8)	
Lower good recovery (7)	427 (7.9)	44 (8.2)	10 (5.3)	
Upper good recovery (8)	514 (9.5)	56 (10.4)	40 (21.4)	
Missing	1270	157	45	

^a^P-value comparing no-NSH and matched NSH patients

^b^Intraventricular catheter for both intracranial pressure monitoring and drainage of cerebral spinal fluid

^c^Invasive monitoring of intracranial pressure includes subdural, subarachnoid, intraparenchymal, intraventricular probe insertion for the sole intracranial pressure monitoring

^d^Neurological complications include intracranial hypertension, episodes of dilated pupils unreactive to light and brain edema

Patients admitted to NSH ICUs experienced a significantly higher number of respiratory (12.9% vs. 5.2%) and infectious (27.7% vs. 19.0%) complications during ICU stay. In contrast, major neurological complications (intracranial hypertension > 20 mmHg, refractory intracranial hypertension, one or more episodes of dilated pupils unreactive to light, and brain edema) were more frequently observed in the no-NSH group (31.9% vs. 25.7%), but without significant difference (*p* = 0.067). When intracranial pressure was not invasively monitored, the diagnosis of intracranial hypertension was based on the CT scan, in the presence of clearly specified signs (reduced or absent cisterns, compressed third or lateral ventricles, evidence of a midline shift in the presence of subfalcine and transtentorial herniation). Nurosurgical interventions performed in NSH centers are provided in Additional file 1: Table S6.

Comparison of GOS-E scores at 6 months post-trauma yielded a significantly different distribution of functional outcomes between the two groups. Patients discharged from no-NSH ICUs more frequently reported good recovery than those discharged from NSH ICUs, while moderate and severe disability were more commonly observed in NSH ICUs. To better explore this finding, the same outcomes were evaluated by stratifying both patient cohorts into severity classes.

**Fig. 3:**
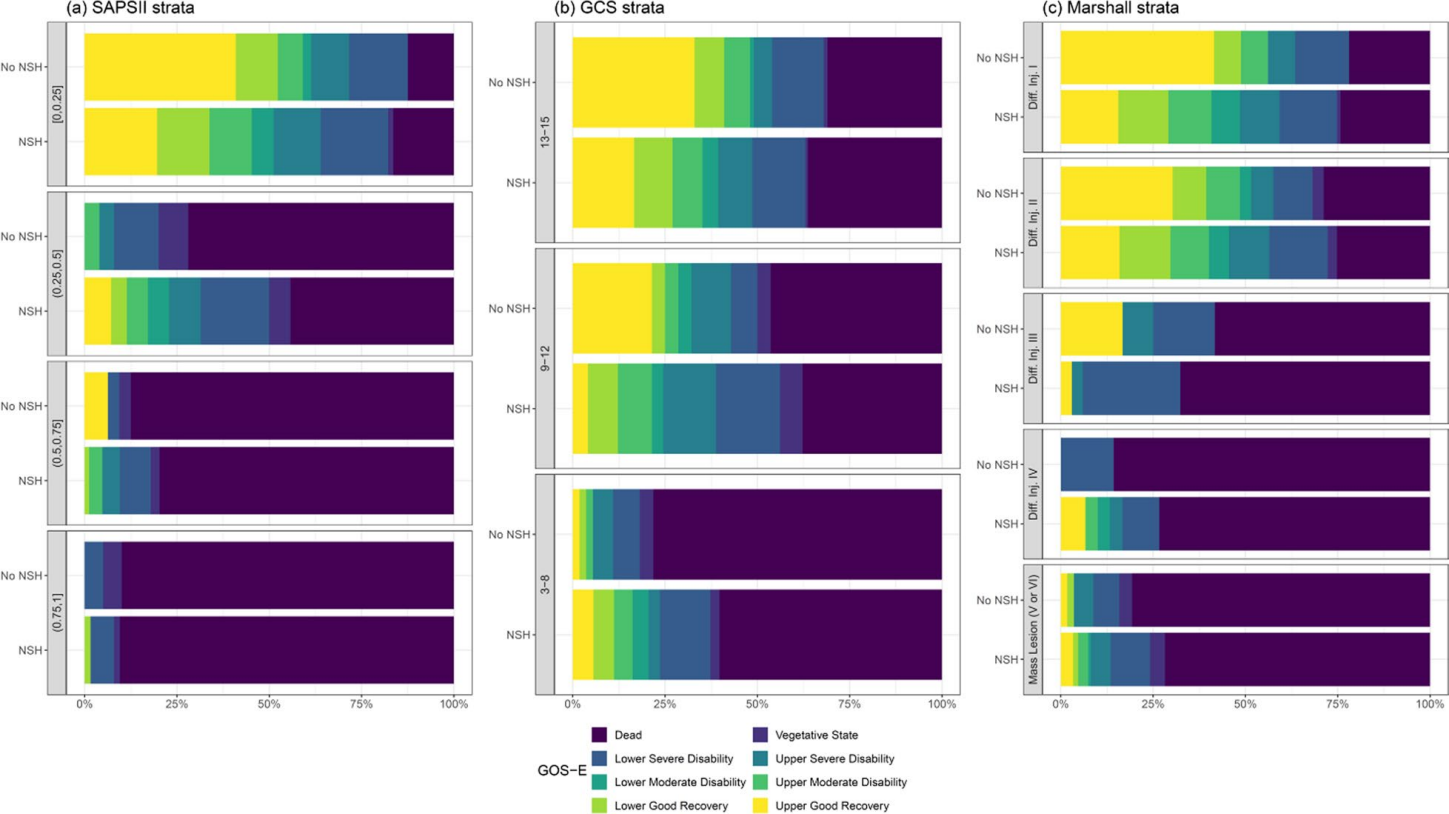
For each SAPSII, GCS and Marshall stratum, the bars compare the distribution of the 6-month GOS-E levels between the NSH and no-NSH groups

Figure 3 shows the distribution of the 6-month GOS-E stratified for predicted mortality at ICU admission, according to SAPS II score, TBI categories based on pre-treatment GCS and the Marshall CT classification. In the stratum with the lowest risk of death according to SAPS II, a higher proportion of good recovery was observed for patients admitted to no-NSH ICUs. The mortality gap between the two groups was at its widest for predicted mortality of 25–50%, with lower mortality in NSH hospitals. The mortality gap narrowed in the group with a 50–75% risk of death, almost disappearing in the highest-risk stratum.

Regarding the TBI and Marshall classification, at 6-month GOS-E, patients treated in no-NSH ICUs showed lower mortality and a higher prevalence of good outcomes in the mild TBI and diffuse injury I groups. Conversely, in moderate and severe TBI patients, death and poor outcomes were significantly more frequent for no-NSH ICUs. Similarly, as the severity of the Marshall classification increased, we observed a progressively higher frequency of worse outcomes for no-NSH patients.

To assess whether our results could have been affected by patients considered in the no-NSH group and secondarily transferred and treated in NSH centers, we quantified the number of secondary transfers from no-NSH centers. Only 11 no-NSH patients (4.7%) were transferred to another ICU for step-up care. Unfortunately, the availability of neurosurgery in the receiving hospital was not collected, nor we collected the information on whether the transfer was motivated by the presence of neurosurgery facilities or for other specialties of the higher-level hospitals. Nonetheless, secondary transfers from no NSH can impact our results only negligibly, as their number is bounded by the very limited number of step-up transfers.

## Discussion

This study focused on TBI patients admitted to ICUs without neurosurgical capabilities, specifically evaluating if their 6-month functional recovery would have been different if these patients had been admitted to an NSH center instead.

Overall, only 3.3% of patients with TBI admitted to an ICU were primarily taken to a hospital without neurosurgery capabilities. The distribution of their severity, based on the SAPS II score, follows a bimodal pattern, yielding a larger cohort of patients with low expected mortality (< 25%) and a smaller group with high expected mortality (50–75%). More than half of the patients were classified as mild TBI; the second most represented group was severe TBI. Older age, low energy impact, higher GCS at ICU admission, and fewer derangements in terms of oxygenation and hemodynamics were more frequent in patients admitted to no-NSH ICUs, as already reported in the literature [26, 27]. These factors, which have been discussed as potential causes of both improper centralization [28, 29] and in-hospital undertriage [30], could, together with distance to higher-level TCs from the trauma site [31] and time of day of the event (in particular for emergency medical helicopter services), have partially contributed to the bimodal distribution of the population with respect to severity score at ICU admission in no-NSH (Fig. 2). Moreover, for the most severely injured patients, transportation to no-NSH centers could also reflect pre-hospital uncertainty about real survival benefits in relation to the transfer time to high-level TCs.

Notably, we found several patients admitted to NSH centers who were similar to those transported to no-NSH ICUs. This was what made the comparison between the two different settings possible, in terms of patients’ outcomes. In this regard, it is essential to point out that we did not evaluate the centralization of TBI patients in general. Instead, we aimed at assessing what would have been the outcome of the TBI patients that were admitted to no-NSH centers had they have been centralized to NSH ones.

The six-month GOS-E distribution showed that patients with the lowest estimated mortality risk admitted to no-NSH ICUs had lower mortality and better functional recovery than similar patients admitted to NSH ICUs. As the risk of death increased, no-NSH ICUs showed higher mortality at 6 months and increasingly poor functional outcomes compared to NSH ICUs. Similarly, when stratifying the cohort by TBI severity, mild TBI patients showed better functional outcomes and lower mortality in no-NSH ICUs, while severe TBI patients showed better functional outcomes and lower mortality in NSH ICUs.

While the results on severe patients are in line with current literature [32, 33], the findings on mild cases are somewhat new and warrant further discussion. Other studies reported better functional outcomes, adjusted for trauma severity scores, in higher-level TCs for the general trauma population [34]. These results are not in disagreement with our findings. While these studies focused on the population of all TBI patients, most of whom are admitted to highly specialized hospitals, our conclusions applies only to those patients (3% of our study cohort) who were admitted to no-NSH centers. On the other hand, a recent study evaluating the TBI population [35] failed to find any effect of centralization to level I TCs in terms of 6-month GOS-E, in a cohort of TBI patients with a median age over 70 years.

One possible explanation for our results is that NSH centers deliver more aggressive and invasive neuro-oriented therapies (see Table 2), and are consequently more focused on these specific problems. On the other hand, no-NSH ICUs may pay greater attention to non-TBI-related clinical aspects, which they are used to manage. For mild TBI patients, these aspects presumably are the most important in determining the outcome. Both hypotheses seem supported by the significantly higher incidence of extra-neurologic complications in NSH centers and the lower, albeit not significant, incidence of major neurologic complications compared to no-NSH ICUs (Table 2). Moreover, the no-NSH centers of our cohort should not be thought as small units in secondary hospitals. On the contrary, they also included high-volume units delivering multiple specialized services, even in the absence of neurosurgery coverage (Additional file 1: Table S4).

The main limitation of this study is its observational nature. The adopted propensity-score matching methodology is a well-established, robust approach that yields an unbiased estimation of causal effects when the underlying assumptions are satisfied. These assumptions include having measured and considered into the analysis all relevant confounders. Our results could be biased if an unobserved prognostic factor has impacted the decision to admit the patient to no-NSH or NSH ICUs, thus leading to an undetectable imbalance of this variable in the two groups of the matched sample. Even though ruling out the existence of unobserved confounders in our study is impossible, it is unlikely that such confounders have severely biased our estimates. Several well-established indicators of severity, related to the severity of brain injury, the baseline conditions and eventual concomitant extracranial traumatic lesions of the patients, have been perfectly balanced in the matched sample. Such indicators include characteristics of the patients measured before the arrival to the hospital and on ICU admission. Moreover, the hypothesis that the overall more favorable 6-month GOS-E of mild patients admitted to no-NSH centers, which is the most original finding of our study, could be due to unobserved confounders, driving undetectably more complex patients to NSH centers, collides with the observed better outcomes of severe TBIs and high-risk patients in NSH centers.

A second significant limitation is NSH and no-NSH centers’ definition, used to classify the individual participating ICUs. Such classification partially limits these findings’ comparability with studies that classify participating facilities by their trauma care level. Neurosurgery capabilities could also be available in level II TCs, which could have partially influenced the observed results [16]. However, classifying the facilities participating in the study by trauma care level would have been difficult because of the heterogeneity of the trauma systems involved in our international initiative. The same challenges in defining a uniform classification across the countries involved in our project also apply to triage criteria. Notably, given the observational nature of our study, each center maintained the same criteria of the trauma system it refers to.

The unavailability of the ISS score in our data is another aspect to discuss. We were not able to calculate such score because we only collected information on the most severe lesions, as defined by an AIS > 2. The ISS score has the advantage of ordinally describing lesion severity in separate districts. However, the strategy adopted for describing trauma-related lesions in our prospective data collection includes precise specification of the location and severity of all the serious individual organ lesions of the AIS dictionary (see Additional file 1: Table S2), often occurring simultaneously in the same district. In order to optimally balance the two populations, individual lesions were considered within the matching procedure and, in our opinion, the resulting balance of the matched cohort was not inferior to what could have been achieved by ISS-based matching. Similar observations apply to specific aspects of TBI, considering that patients were matched on numerous variables, including specific intracranial lesions and related volume, Marshall scale, GCS score, and pupil reactivity.

Most ICUs participating in this study were based in Italy (67.5%, see Additional file 1: Table S4) and, in particular, the proportion of Italian ICUs among no-NSH centers was higher than in the NSH group. Moreover, the characteristics of the no-NSH hospitals in our study may differ from those of the no-NSH centers in other systems. For these reasons, our results should be generalized with caution to very different contexts.

Finally, socioeconomic and rehabilitative factors could have played a role in determining final functional outcome but, due to the high frequency of missing data, these variables were not taken into consideration.

To overcome the limitations of our study and verify the generalizability of its findings, further research is essential. Given the logistic and ethical challenges of randomized trials in this context, such verification needs to rely on specifically designed, prospective observational studies. In particular, to verify whether patients admitted to an ICU in no-NSH hospitals would benefit from treatment in NSH centers, the envisioned study must satisfy two properties. First, the data collection should be carefully planned, to include all variables that may both impact on the outcome of the patient and on the decision of admitting a patient to a NSH or a no-NSH hospital. This is essential to apply rigorous statistical methodology to estimate causal effects on observational data and minimize the risk of unmeasured confounding. Second, the recruitment of the study should aim at minimizing differences in terms of country and even of trauma systems between NSH and no-NSH centers, to minimize the confounding effect of the different contexts where ICUs operate.

## Conclusion

For patients admitted to ICUs without neurosurgical capabilities, we found that admission to NSH centers may be beneficial, in terms of increased survival and higher percentage of good recovery, in the subgroup of patients with severe TBI or at high risk according to the SAPS II score. Conversely, admission to no-NSH centers was associated to higher rates of good recovery and lower mortality in mild TBI and low-risk patients. This unexpected result could have been partially conditioned by greater attention to the management of non-neurologic clinical conditions in no-NSH ICUs, by the adverse effects of more aggressive neuro-oriented therapies performed in the NSH centers, or by the presence of confounders not taken into consideration in the matching procedure.

All in all, this study underlines how the triage of TBI patients to hospitals with different neurosurgical capabilities and facilities could have a marked effect on middle- and long-term outcomes.

## Supplementary Information

**Additional file 1.** The additional file provides supplementary tables and figures that could not fit in the paper.

## Data Availability

The datasets used and/or analysed during the current study are available from the corresponding author on reasonable request.
