# Supplementary material for: Hospitals with and without neurosurgery: a comparative study evaluating the outcome of patients with traumatic brain injury
Source: Scand J Trauma Resusc Emerg Med. 2021 Nov 2;29:158. doi: 10.1186/s13049-021-00959-2 (PMC8561979; doi:10.1186/s13049-021-00959-2)

**Additional file 1 of**

**“Hospitals with and without neurosurgery: A comparative study evaluating the outcome of patients with traumatic brain injury”**

**Table S.1.** List of comorbidities collected.

| Area | Comorbidity |
| --- | --- |
| Respiratory | Asthma  Moderate COPD  Severe COPD  Restrictive lung disease |
| Cardiovascular | Arrhythmia  Myocardiopathy  Heart failure (NYHA class II-III or ACC stage C)  Heart failure (NYHA class IV or ACC stage D)  Myocardial infarction  Hypertension  Peripheral vascular disease |
| Neurological | Dementia  Hemiplegia or paraplegia or quadriplegia  Cerebrovascular disease  Neurodegenerative/Neuromuscular disease |
| Gastrointestinal and hepatic | Peptic ulcer disease  Mild liver disease  Moderate or severe liver disease |
| Renal | Moderate or severe renal disease  End-stage renal disease |
| Endocrine | Diabetes Type I  Diabetes Type II with insulin treatment  Diabetes Type II without insulin treatment |
| Malignancy | Any tumour without metastasis  Metastatic cancer  Malignant haematological disease |
| Other | Autoimmune disease  Immunosuppression  Drug-induced coagulopathy  Coagulation disorder  Antiplatelet therapy  AIDS  Severe malnutrition |

**Table S.2.** List of lesions considered in each body region.

| Body region | Lesion |
| --- | --- |
| Spine | Cervical spinal cord injury with tetraplegia  Cervical spinal cord injury with incomplete neurologic deficit  Dorsal spinal cord injury with paraplegia  Dorsal spinal cord injury with incomplete neurologic deficit  Lumbar spinal cord injury with complete neurologic deficit  Lumbar spinal cord injury with incomplete neurologic deficit  Vertebral fracture without neurologic deficit |
| Chest | Major laceration of trachea/larynx  Oesophagus: rupture/perforation  Traumatic haemothorax and/or pneumothorax  Traumatic massive haemothorax  Tension pneumothorax  Flail chest  Severe lung contusion/laceration  Cardiac trauma  Diaphragmatic rupture  Other injuries of the chest |
| Abdomen | Stomach: Rupture or perforation  Bowel: Complete transection or perforation  Pancreas: Laceration  Liver: Moderate-Severe laceration  Liver: Massive laceration  Spleen: Moderate-Severe laceration  Spleen: Massive rupture  Kidney: Rupture/laceration  Minor injuries of the abdomen |
| Pelvis, bones, joints and muscles | Long bone fracture  Very severe or open fracture of the pelvis with unstable pelvis  Multiple fracture of the pelvis  Massive crush/amputation of one or more limbs  Extremity compartment syndrome |
| Major vessels | Aorta: rupture/dissection  Cava: rupture/transection  Major thoracic vessels: transection  Major abdominal vessels: transection  Major vessels of the neck: dissection/transection  Major vessels of the proximal limbs: transection |
| Other | Burns (> 30% of total body surface area)  Inhalation injury |

**Table S.3.** Estimates and 95% confidence intervals of the odds ratio of the propensity score model.

| Variable | Odds Ratio | 95% CI - Lower | 95% CI - Upper |
| --- | --- | --- | --- |
| Age (10-year increase) | 1.25 | 1.16 | 1.36 |
| Dementia | 0.80 | 0.33 | 1.66 |
| Drug-induced coagulopathy | 1.07 | 0.62 | 1.74 |
| Antiplatelet therapy | 0.84 | 0.50 | 1.34 |
| Haemorrhagic-Hypovolemic shock | 0.53 | 0.25 | 1.01 |
| Neurogenic shock | 0.82 | 0.39 | 1.53 |
| Liver: massive rupture or moderate-severe laceration | 0.51 | 0.08 | 1.72 |
| Spleen: massive rupture or moderate-severe laceration | 0.26 | 0.04 | 0.87 |
| Very severe or open fracture of the pelvis with unstable pelvis | 0.48 | 0.03 | 2.51 |
| Spinal cord injury | 0.57 | 0.14 | 1.60 |
| Main lesion (Cerebral contusion/laceration vs. Skull fracture) | 0.78 | 0.29 | 2.40 |
| Main lesion (Extradural/epidural haematoma vs. Skull fracture) | 0.79 | 0.25 | 2.71 |
| Main lesion (Traumatic subdural haematoma vs. Skull fracture) | 0.95 | 0.36 | 2.93 |
| Main lesion (Intraparenchymal bleeding vs. Skull fracture) | 3.18 | 1.19 | 9.86 |
| Main lesion (Diffuse injury without oedema vs. Skull fracture) | 0.56 | 0.14 | 2.11 |
| Main lesion (Diffuse injury with oedema vs. Skull fracture) | 1.38 | 0.34 | 5.43 |
| Main lesion (Subarachnoid haemorrhage vs. Skull fracture) | 1.71 | 0.75 | 4.64 |
| Type of trauma (Penetrating vs. Closed) | 0.54 | 0.16 | 1.34 |
| Traffic accident | 0.81 | 0.59 | 1.10 |
| Pre-treatment GCS (3-6 vs. 15) | 0.87 | 0.50 | 1.49 |
| Pre-treatment GCS (7-8 vs. 15) | 0.42 | 0.20 | 0.84 |
| Pre-treatment GCS (9-10 vs. 15) | 0.78 | 0.41 | 1.44 |
| Pre-treatment GCS (11-12 vs. 15) | 0.95 | 0.52 | 1.70 |
| Pre-treatment GCS (13-14 vs. 15) | 0.95 | 0.61 | 1.47 |
| GCS on ICU admission (3-6 vs. 15) | 0.40 | 0.23 | 0.69 |
| GCS on ICU admission (7-8 vs. 15) | 0.29 | 0.14 | 0.55 |
| GCS on ICU admission (9-10 vs. 15) | 0.40 | 0.21 | 0.76 |
| GCS on ICU admission (11-12 vs. 15) | 0.36 | 0.17 | 0.71 |
| GCS on ICU admission (13-14 vs. 15) | 0.59 | 0.35 | 0.97 |
| GCS on ICU admission (Not evaluable vs. 15) | 0.12 | 0.06 | 0.22 |
| Pupils (Unilaterally dil./non-reactive vs. Bilaterally react./miotic) | 0.86 | 0.49 | 1.45 |
| Pupils (Bilaterally dil./non-reactive vs. Bilaterally react./miotic) | 0.85 | 0.40 | 1.67 |
| Pupils (Not available vs. Bilaterally react./miotic) | 1.44 | 0.59 | 3.06 |
| Hypotension | 0.91 | 0.53 | 1.50 |
| Hypoxia | 0.47 | 0.29 | 0.72 |
| Marshall (Diff. Inj. II vs. Diff. Inj. I) | 1.09 | 0.40 | 3.03 |
| Marshall (Diff. Inj. III vs. Diff. Inj. I) | 0.45 | 0.15 | 1.27 |
| Marshall (Diff. Inj. IV vs. Diff. Inj. I) | 1.23 | 0.35 | 4.14 |
| Marshall (Mass Lesion V or VI vs. Diff. Inj. I) | 0.32 | 0.09 | 1.00 |
| Midline shift>5 mm | 0.71 | 0.40 | 1.30 |
| Cistern conditions (Compressed or distorted vs. Normal) | 1.70 | 0.90 | 3.39 |
| Cistern conditions (Absent vs. Normal) | 1.24 | 0.50 | 3.06 |
| Petechiae | 1.02 | 0.72 | 1.42 |
| Lesion volume > 25ml | 1.37 | 0.66 | 3.21 |

**Table S.4.** Characteristics of the participating ICUs.

| Variables | NSH | | No NSH |
| --- | --- | --- | --- |
|  | All | Matched |  |
| N ICUs | 66 | 53 | 14 |
| Country – N (%) |  |  |  |
| Cyprus | 1 (1.5) | 1 (1.9) | 0 (0.0) |
| Greece | 6 (9.1) | 3 (5.7) | 0 (0.0) |
| Hungary | 7 (10.6) | 6 (11.3) | 0 (0.0) |
| Israel | 2 (3.0) | 0 (0.0) | 0 (0.0) |
| Italy | 41 (62.1) | 36 (67.9) | 13 (92.9) |
| Poland | 5 (7.6) | 4 (7.5) | 1 (7.1) |
| Slovenia | 4 (6.1) | 3 (5.7) | 0 (0.0) |
| Vascular surgery – N (%) | 60 (92.3) | 48 (90.6) | 8 (57.1) |
| Missing | 1 | 0 | 0 |
| Interventional vascular radiology – N (%) | 60 (92.3) | 48 (90.6) | 7 (50.0) |
| Missing | 1 | 0 | 0 |
| CT scan – N (%) | 66 (100.0) | 53 (100.0) | 14 (100.0) |
| Missing | 0 | 0 | 0 |
| MRI – N (%) | 64 (98.5) | 52 (98.1) | 10 (71.4) |
| Missing | 1 | 0 | 0 |
| Hospital beds – N (%) |  |  |  |
| Mean (SD) | 776.5 (434.9) | 792.6 (453.8) | 328.2 (124.6) |
| Median (Q1-Q3) | 675 (473 – 1000) | 664 (473 – 1048) | 333 (215 – 397) |
| Missing | 2 | 1 | 0 |
| ICU beds – N (%) |  |  |  |
| Mean (SD) | 11.0 (4.1) | 11.2 (4.2) | 6.6 (2.4) |
| Median (Q1-Q3) | 10 (8 – 13) | 10 (8 – 13) | 6 (5 – 7) |
| Missing | 2 | 0 | 0 |
| Occupancy rate – N (%) |  |  |  |
| Mean (SD) | 77.2 (14.9) | 77.9 (15.2) | 67.7 (11.4) |
| Median (Q1-Q3) | 78.6 (68.7 –88.9) | 78.8 (69.2 – 89.8) | 68.3 (61.2 – 75.6) |
| Missing | 5 | 3 | 0 |
| Number of beds/physician – N (%) |  |  |  |
| Mean (SD) | 4.2 (1.5) | 4.2 (1.3) | 5.1 (1.6) |
| Median (Q1-Q3) | 3.9 (3.2 – 5.0) | 3.9 (3.2 – 5.0) | 4.9 (4.0 – 6.2) |
| Missing | 2 | 0 | 0 |
| Number of beds/nurse – N (%) |  |  |  |
| Mean (SD) | 2.1 (0.4) | 2.1 (0.4) | 2.3 (0.3) |
| Median (Q1-Q3) | 2.1 (1.9 – 2.4) | 2.1 (1.9 – 2.4) | 2.2 (2.0 – 2.6) |
| Missing | 3 | 0 | 0 |
| Number of trauma/year – N (%) |  |  |  |
| Mean (SD) | 89.4 (62.1) | 97.0 (63.8) | 28.4 (12.6) |
| Median (Q1-Q3) | 73.0 (45.9 – 107.2) | 77.9 (54.8 – 115.3) | 28.0 (25.6 – 34.9) |
| Missing | 6 | 5 | 2 |
| Number of traumatic brain injury/year – N (%) |  |  |  |
| Mean (SD) | 51.9 (33.1) | 56.4 (33.6) | 8.8 (5.1) |
| Median (Q1-Q3) | 45.0 (29.7 – 66.0) | 47.6 (31.8 – 67.7) | 9.8 (4.0 – 10.8) |
| Missing | 6 | 5 | 2 |

**Table S.5.** Absolute standardized mean differences (ASD) for the propensity score variables.

| Variable | ASD  Before Matching | ASD  After Matching |
| --- | --- | --- |
| Age | 0.46 | 0.00 |
| Dementia | 0.02 | 0.06 |
| Drug-induced coagulopathy | 0.12 | 0.04 |
| Antiplatelet therapy | 0.03 | 0.02 |
| Haemorrhagic-hypovolemic shock | 0.24 | 0.01 |
| Neurogenic shock | 0.15 | 0.02 |
| Liver: massive rupture or moderate-severe laceration | 0.14 | 0.00 |
| Spleen: massive rupture or moderate-severe laceration | 0.22 | 0.01 |
| Very severe or open fracture of the pelvis with unstable pelvis | 0.08 | 0.02 |
| Spinal cord injury | 0.09 | 0.04 |
| Main lesion: Cerebral contusion/laceration | 0.21 | 0.02 |
| Main lesion: Extradural/epidural haematoma | 0.18 | 0.07 |
| Main lesion: Traumatic subdural haematoma | 0.17 | 0.05 |
| Main lesion: Intraparenchymal bleeding | 0.35 | 0.01 |
| Main lesion: Diffuse injury without oedema | 0.21 | 0.03 |
| Main lesion: Diffuse injury with oedema | 0.13 | 0.02 |
| Main lesion: Subarachnoid haemorrhage | 0.38 | 0.03 |
| Main lesion: Skull fracture | 0.03 | 0.04 |
| Type of Trauma (Penetrating vs. Closed) | 0.09 | 0.04 |
| Traffic Accident | 0.19 | 0.01 |
| Pre-treatment GCS: 3 | 0.21 | 0.02 |
| Pre-treatment GCS: 4 | 0.15 | 0.01 |
| Pre-treatment GCS: 5 | 0.11 | 0.04 |
| Pre-treatment GCS: 6 | 0.07 | 0.01 |
| Pre-treatment GCS: 7 | 0.24 | 0.00 |
| Pre-treatment GCS: 8 | 0.19 | 0.01 |
| Pre-treatment GCS: 9 | 0.01 | 0.05 |
| Pre-treatment GCS: 10 | 0.08 | 0.06 |
| Pre-treatment GCS: 11 | 0.01 | 0.01 |
| Pre-treatment GCS: 12 | 0.01 | 0.09 |
| Pre-treatment GCS: 13 | 0.05 | 0.08 |
| Pre-treatment GCS: 14 | 0.22 | 0.10 |
| Pre-treatment GCS: 15 | 0.39 | 0.06 |
| GCS on ICU admission: 3 | 0.11 | 0.06 |
| GCS on ICU admission: 4 | 0.00 | 0.03 |
| GCS on ICU admission: 5 | 0.07 | 0.05 |
| GCS on ICU admission: 6 | 0.09 | 0.08 |
| GCS on ICU admission: 7 | 0.14 | 0.02 |
| GCS on ICU admission: 8 | 0.11 | 0.03 |
| GCS on ICU admission: 9 | 0.04 | 0.04 |
| GCS on ICU admission: 10 | 0.00 | 0.07 |
| GCS on ICU admission: 11 | 0.02 | 0.07 |
| GCS on ICU admission: 12 | 0.05 | 0.05 |
| GCS on ICU admission: 13 | 0.13 | 0.04 |
| GCS on ICU admission: 14 | 0.24 | 0.04 |
| GCS on ICU admission: 15 | 0.52 | 0.07 |
| GCS on ICU admission not assessable | 0.54 | 0.02 |
| Pupils at ED arrival: Bilaterally reactive and/or miotic | 0.31 | 0.01 |
| Pupils at ED arrival: Unilaterally dilated and non-reactive | 0.23 | 0.01 |
| Pupils at ED arrival: Bilaterally dilated and non-reactive | 0.19 | 0.02 |
| Pupils at ED arrival: Not available | 0.02 | 0.02 |
| Clinically relevant hypotension | 0.21 | 0.00 |
| Clinically relevant hypoxia | 0.36 | 0.01 |
| Marshall scale: Diffuse Injury I | 0.34 | 0.02 |
| Marshall scale: Diffuse Injury II | 0.09 | 0.05 |
| Marshall scale: Diffuse Injury III | 0.15 | 0.02 |
| Marshall scale: Diffuse Injury IV | 0.07 | 0.01 |
| Marshall scale: Mass Lesion (V or VI) | 0.31 | 0.05 |
| Midline shift>5 mm | 0.23 | 0.05 |
| Cistern conditions: Normal | 0.01 | 0.07 |
| Cistern conditions: Compressed or distorted | 0.19 | 0.06 |
| Cistern conditions: Absent | 0.23 | 0.02 |
| Petechiae | 0.23 | 0.01 |
| Lesion volume > 25ml | 0.22 | 0.04 |

**Table S.6.** Neurosurgical procedures performed in NSH centers. Missing values are due to the start of data collection on neurosurgical procedures at different timings during the course of the study.

| Variables | NSH | |
| --- | --- | --- |
|  | All | Matched |
| N patients | 6,682 | 696 |
| Subdural haematoma evacuation |  |  |
| N (%) | 1,239 (20.4) | 79 (12.0) |
| Missing | 618 | 37 |
| Extradural haematoma evacuation |  |  |
| N (%) | 360 (5.9) | 17 (2.6) |
| Missing | 619 | 37 |
| Lobectomy or contusion removal |  |  |
| N (%) | 181 (3.0) | 18 (2.7) |
| Missing | 619 | 37 |
| Primary decompression |  |  |
| N (%) | 694 (10.4) | 33 (4.7) |
| Missing | 16 | 1 |
| Secondary decompression |  |  |
| N (%) | 176 (2.6) | 11 (1.6) |
| Missing | 16 | 1 |

**Figure S.1.** Distribution of subjects across centers in the NSH group (panel (a)), matched NSH group (panel (b)) and no-NSH group (panel (c)).





**Figure S.2.** The two panels compare the distribution of the logit of the propensity score between the NSH and no-NSH groups before matching (upper panel) and after matching (bottom panel). The area under the curves corresponds to the size of the corresponding group.


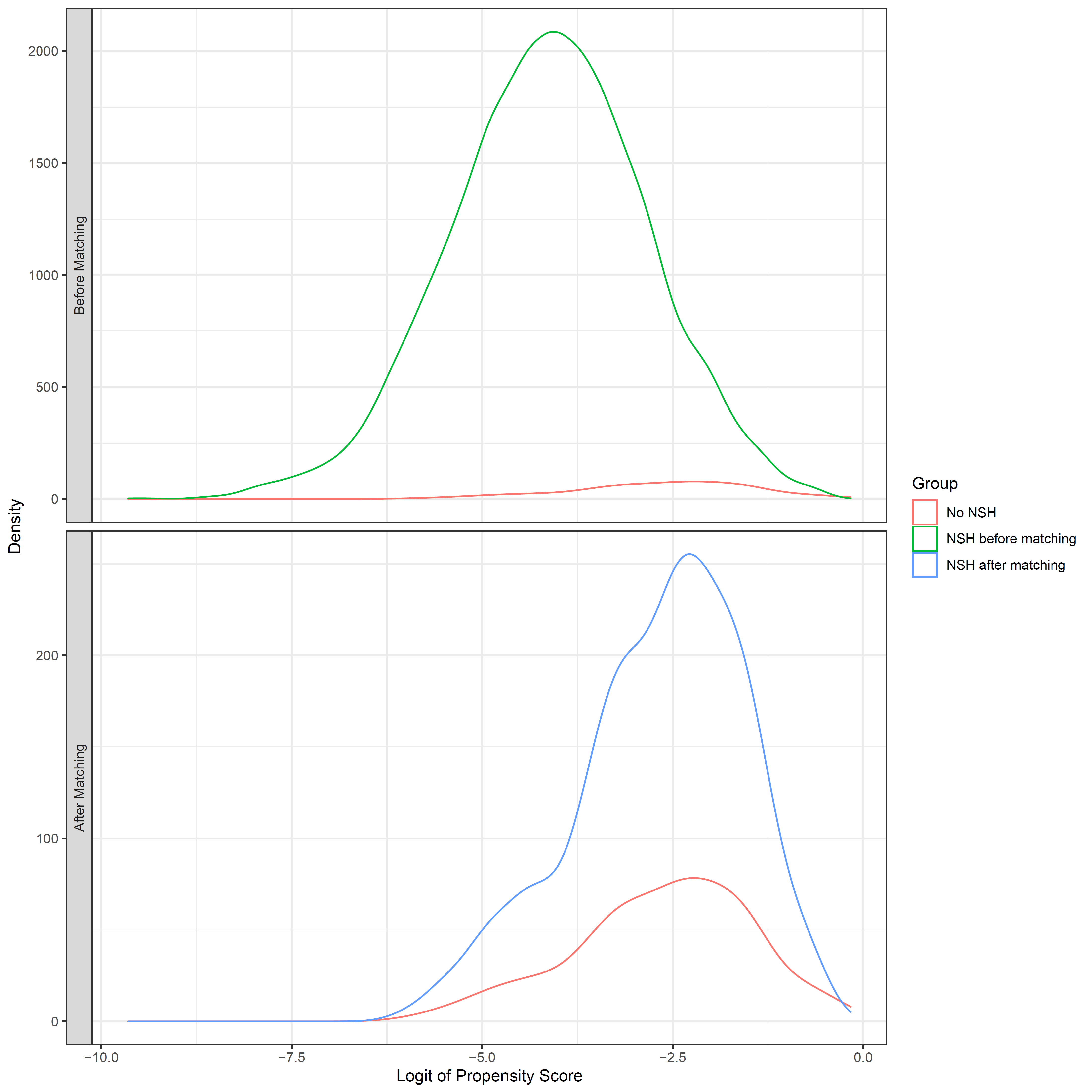

Supplement: Supplementary file 1 — Additional file 1. The additional file provides supplementary tables and figures that could not fit in the paper. [file 13049_2021_959_MOESM1_ESM.docx]
